# Clinical outcomes and characteristics of P30L mutations in congenital adrenal hyperplasia due to 21-hydroxylase deficiency

**DOI:** 10.1007/s12020-020-02323-3

**Published:** 2020-05-05

**Authors:** Mirjana Kocova, Violeta Anastasovska, Henrik Falhammar

**Affiliations:** 1 Medical Faculty, University“Cyril&Methodius”, Skopje, Republic of North Macedonia; 2 Genetic Laboratory, University Pediatric Hospital, Skopje, Republic of North Macedonia; 3 Department of Endocrinology, Metabolism and Diabetes, Karolinska University Hospital, Stockholm, Sweden; 4 Departement of Molecular Medicine and Surgery, Karolinska Institutet, Stockholm, Sweden

**Keywords:** Nonclassic, Simple virilizing, P30L, *CYP21A2*, Diagnosis, Therapy

## Abstract

Despite numerous studies in the field of congenital adrenal hyperplasia (CAH) due to 21-hydroxylase deficiency, some clinical variability of the presentation and discrepancies in the genotype/phenotype correlation are still unexplained. Some, but not all, discordant phenotypes caused by mutations with known enzyme activity have been explained by in silico structural changes in the 21-hydroxylase protein. The incidence of P30L mutation varies in different populations and is most frequently found in several Central and Southeast European countries as well as Mexico. Patients carrying P30L mutation present predominantly as non-classical CAH; however, simple virilizing forms are found in up to 50% of patients. Taking into consideration the residual 21-hydroxulase activity present with P30L mutation this is unexpected. Different mechanisms for increased androgenization in patients carrying P30L mutation have been proposed including influence of different residues, accompanying promotor allele variability or mutations, and individual androgene sensitivity. Early diagnosis of patients who would present with SV is important in order to improve outcome. Outcome studies of CAH have confirmed the uniqueness of this mutation such as difficulties in phenotype classification, different fertility, growth, and psychologic issues in comparison with other genotypes. Additional studies of P30L mutation are warranted.

## Introduction

Congenital adrenal hyperplasia (CAH) is a family of autosomal recessive disorders caused by mutations of genes involved in the steroidogenesis pathway [1–4]. The most common cause of CAH, occurring in 95–99% of cases, is 21-hydroxylase deficiency (21OHD), followed by 11β-hydroxylase deficiency, 17α-hydroxylase/17,20-lyase deficiency, 3β-hydroxy-steroid dehydrogenase type 2 deficiency, P450 oxidoreductase deficiency, lipoid CAH, and cholesterol side chain cleavage enzyme deficiency [4–10]. Mutations in the cytochrome P450 (CYP) 21A2 (*CYP21A2*) gene result in 21OHD. The cytochromes P450 comprise a superfamily of heme-containing mono-oxygenases that play central roles in the metabolism of a wide variety of endogenous compounds including steroids, drugs, and carcinogens.

CAH can clinically be manifested in a variety of forms, depending on the amount of the functioning enzyme. The common classification consists of two major forms, classical and nonclassical [1, 11]. Classical form of 21OHD appears with an incidence of 1:10,000–1:20,000 live births in different populations [4, 12–14], and is rising with the early detection by neonatal screening [15]. The phenotype of patients with a classical 21OHD is different depending on the degree of the remaining 21-hydroxylase enzyme activity. In a majority of patients with classical 21OHD (75%), 21-hydroxylase activity is completely absent causing life-threatening cortisol and aldosterone deficiency (salt-wasting form [SW]) accompanied with hyperandrogenemia causing sexual ambiguity in affected females [2, 16, 17]. If the remaining enzyme activity is low but present (<2%), simple virilizing (SV) form of the disease occurs which appears in 25% of cases with the classical form. SV is characterized by a cortisol deficiency accompanied with hyperandrogenemia inducing virilization of the external genitalia in females and hyperandrogenemia in boys, often noticed by precocious puberty. The nonclassical form of 21OHD (NC) is common, one of the most common recessive disease in humans (1/200–1/1000 in Caucasians), especially in certain ethnicities such as Eastern European Jews, Finland being an exception with the lowest incidence [18–22]. The enzyme activity in NC CAH is preserved (~20–60%) with symptoms appearing later in life, mostly in preadolescent, adolescent or young adult period [20, 23, 24]. Oligoamenorrhea, hirsutism, and impaired fertility are common symptoms.

The *CYP21A2* gene is located on chromosome 6.21p in the region of class III of the human leukocyte antigen [25]. Several other genes are located in this region forming a module. *CYP21A2* is located 30 kb upstream of its nonfunctional pseudogene *CYP21A1* [26]. Both genes consist of ten exons, and have high sequence homology (98% exonic nucleotide homology and 96% intronic homology) [7, 26, 27]; however, the pseudogene is nonfunctional. Gene changes comprise large deletions, 8 bp deletions, gene conversions due to crossing-over with the adjacent pseudogene, and point mutations in the gene itself [20, 28]. The pseudogene is prone to mutations (e.g., splicing, frameshift, and insertions) which can be transferred to the functional gene by microconversion events. Most frequently *CYP21A2* mutations occur as a result of recombination with the pseudogene (75%). The remaining 20–25% of mutations consist of deletions or chimeric genes, both appearing as a result of unequal crossing-over. Only 1% of mutations appear de novo [1]. The number of reported *CYP21A2* mutations increases continuously from around 130 [28–32] to over 230 in the last large reports [33, 34]. Most patients with 21OHD are compound heterozygotes carrying different mutated alleles. In consanguineous populations, homozygocity is more common compared with admixed populations. Specific expression variations might also be a problem such as the leaky intron 2 mutation and alternative splicing or duplication of Q318X allele [35, 36].

Mutations are classified as severe, moderate, and mild based upon the enzyme activity and the phenotype they most frequently induce. The phenotype depends on the activity of the milder mutation in compound heterozygotes since it is connected with some enzyme activity [24, 33, 37].

P30L has been classified to the group of the milder mutations with 20–60% of enzyme activity [1, 38]. Most frequently, P30L mutation causes mild, i.e., NC pheontype. However, it can also cause SV form with intensive hyperandrogenism and virilization [20, 22, 39–41]. The P30L mutation frequency in different populations and the clinical outcomes in patients carrying P30L mutation have not been extensively reviewed.

The aim of this review is to present clinical, including long-term, outcomes, and molecular findings associated with the P30L mutation.

## Molecular structure of P30L mutation and functional analysis

Molecular characterization of the *CYP21A2* mutations and their impact upon the structure of the 21-hydroxylase enzyme influence the phenotype and the severity of the disease [7, 31, 39, 42–45]. The human crystal structure model of the 21-hydroxylase enzyme has been unraveled recently and the impact of numerous different mutations has been explored, explaining the SW, SV, and NC phenotypes [31, 46, 47]. The CYP21A2 molecule resides in the membranes of endoplasmatic reticulum. Different chaperons and cellular proteins assist the proper folding of the protein. The enzyme has a triangular prism shape and contains 16 helices and 9 β-sheets with a heme located centrally [31]. There are two binding sites for 17-hydroxyprogesterone (17OHP), proximal and distal, both in the proximity of the heme moiety. Hydrogen bonds of residues connected to the heme, as well as the electron transfer are crucial for the proper function of the enzyme [31, 33, 47]. Mutations which cause irregular clashes with heme of the enzyme, disruption of hydrogen bonds, substrate binding, mutations causing impaired secondary structure or structural stability, all abrogate the enzyme function and cause the severe SW form of the disease [31, 46, 48–50]. On the other hand, mutations causing only reduction in the enzyme anchoring, but allow some residual function, cause SV or NC 21OHD. Moreover, interruption of inter- and intra-protein interactions or hydrophobic environment disruption may cause unexpected phenotypes. Thus I172N can occasionally cause NC, and V281L can be found rarely in SW form [31, 51].

P30L mutation in exon 1 of *CYP21A2* gene is a mild missense single base pair mutation (g.89 C>T), which belongs to the pseudogene-derived mutations. It decreases 21-hydroxylase activity to 20–60% according different authors [1, 38, 52], frequently causing NC 21OHD. It is not associated with particular HLA antigens in contrast to V281L which is associated with the haplotype B14;DR1 [1].

**Fig. 1:**
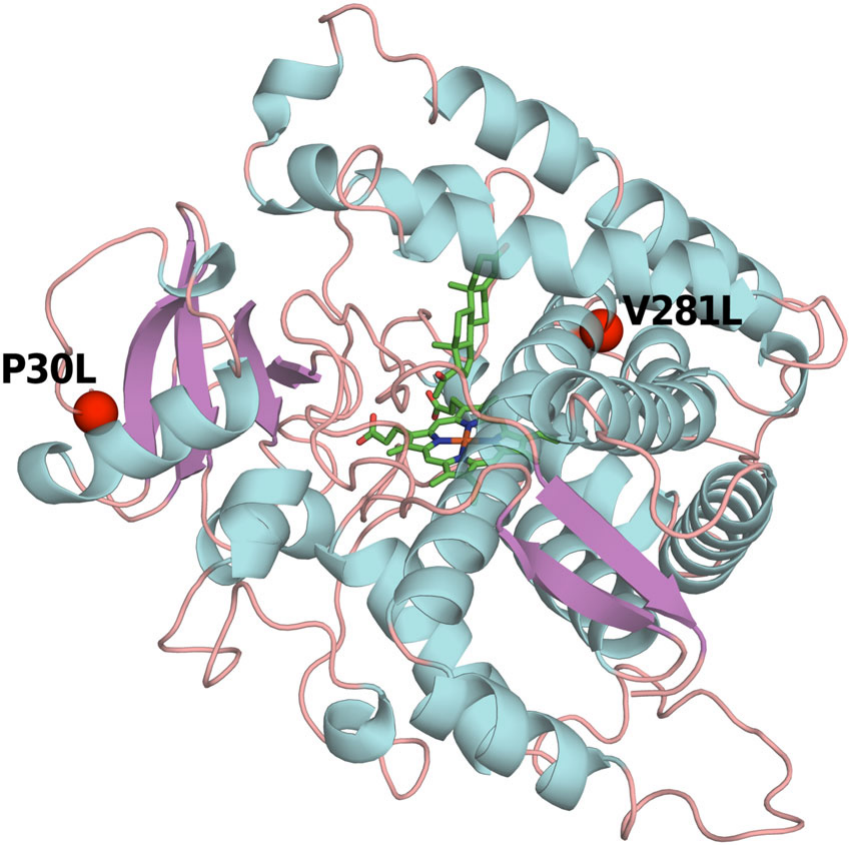
Spatial localization of the mutant residues P30L and V281L in the CYP21A2 protein showing their peripheral localization. P30L is localized at the N terminus of the protein responsible for the orientation toward the membrane of the endoplasmatic reticulum

P30 residue is located at the N-terminal site of the enzyme near to the transmembrane region. It is lodged in a hydrophobic cavity of the enzyme and is crucial for attachment of P450 to the membrane. When prolin is replaced with glutamin (P30Q g89 C>A) its hydrophilic properties disrupt the hydrophobic network and affects the attachment of the enzyme to the membrane with SW form as a result. Replacement of prolin with leucine (P30L g89 C>T), which is hydrophobic residue, interferes with the proper orientation of the enzyme with respect to the microsomal membrane segment, orienting the protein away from membrane, but not improper folding of the protein. Therefore P30L mutation is better tolerated [46, 53]. Analyzing the protein stability of P30L mutation has shown that the half-life is significantly reduced compared with other mild mutations [31, 54]. Enzymes carrying P30L mutations were initially structurally studied in detail and confirmed as a cause of NC form in 1991 [53]. The authors used recombinant vaccine virus to express two mutant enzymes carrying P30L as a pathologic mutation and Ser268Thr conferring normal polymorphism as a first control. The wild type of the enzyme was used as a second control. The normal polymorphism showed 100% enzyme activity, the same as the wild type, whereas, the enzyme activity of P30L mutation was 60% for 17OHP and only 25% for binding progesterone as a substrate. The speed of metabolism affected by P30L mutation for 17OHP and progesterone compared with the wild type was 12- and 21-fold lower, respectively [53]. Furthermore, enzymatic activity with P30L mutation was rapidly lost when the cells were lyzed, suggesting relative enzyme instability [1]. Using computational methods Neves Cruz et al. evaluated the structural impact and the effect on the steroid binding as well as protein structural conformation of different *CYP21A2* mutations [47]. P30L mutation is located peripherally at the N end of the enzyme and is involved in moderate change of enzyme stability (Fig. 1). However, it showed conserved metallic coordination between the heme group and the Cys428 residue of the polypeptide chain with an average distance of 2.5 Å which is similar with the wild type. This is pivotal for the preservation of the P450 enzymes activity, therefore P30L mutation is frequently associated with the NC phenotype [47]. In a study by Tussie-Luna et al. the mutation was present in 28% (5/18) of patients with hormonal evidence of NC CAH. However, all patients carrying the P30L mutation were symptomatic, in contrast to 69% (9/13) displaying other NC mutations. It is worth mentioning that four out of five patients with P30L had clitoromegaly compared with none of those carrying other NC mutations (e.g., V281L) [53]. Clitoromegaly was also reported in NC patients carrying P30L in China [55]. This shows that the P30L mutation, although the enzyme activity is preserved sufficiently, causes a more severe form of NC or even a SV form. E.g., in one family, the proband had P30L/I172N, and presented as SV form with clitoromegaly, hirsutism, delayed menarche, and severely impaired fertility [40].

It is still not known whether in certain patients with P30L mutation other residues are unfavorable, and the enzyme, although present, cannot function appropriately causing increased virilization corresponding to SV CAH [31]. Some authors believe that decreased activity in P30L is due to amino acid residues affecting other functions such as heme coordination, posttranslational modifications, or interface with other interacting proteins or ligands [32, 53]. The P30L mutation obviously has additional regulators, or posttranslational inhibitors of enzyme activity. Some additional residues might also have influence [33]. Simultaneous transfer of mutation in the promotor region together with the P30L mutation from the pseudogene has been described causing a fivefold decrease of the gene activity and causing SV form [56]. Moreover, different allele variations in the promotor region of *CYP21A2* gene causing 50% lower transcriptional activity of 21-hydroxylase have been identified in SV patients carrying P30L mutation [57]. The presence of unidentified rare mutations modulating the phenotype cannot be excluded; however, it is unlikely due to the frequent occurrence of SV form with P30L mutation in studies with meticulous genotyping. Some other factors modifying phenotype have also been suggested such as the CAG repeats of the androgen receptor and other genes encoding proteins other than cytochrome P450 type II enzyme with a 21-hydroxylase activity, as well as alternative pathway of androgen biosynthesis causing fetal virilization in females [58–60].

## Prevalence of P30L mutation

The frequency of different *CYP21A2* mutations are variable in different populations and ethnicities [7, 16, 45, 61–63]. The most common mutations are deletion or large gene conversion, I2 splice, V281L, and I172N [7, 14, 16, 37, 45]. In populations with consanguinity the variability is lower compared with admixed populations [64, 65]. Different reports show that the P30L is not among the most common mutations. Its frequency is between 0 and 46% in different populations (Table 1) [14, 16, 18, 19, 23, 37, 45, 55, 62, 63, 66–78]. Interestingly, P30L mutation occur more frequently in Central and Southeast Europe, including Balkans, and Mexico [39, 62, 63, 68, 74, 79, 80].

**Table 1:** Prevalence of P30L and V281L mutations in different populations and corresponding phenotypes

Country	Number of alleles	P30L	Form of CAH	V281L	Form of CAH	Reference
Argentina	908	0.9%	NC, SV	26.2%	NC	Marino et al. [16]
Brazil	960	0.6%	SV, NC	26.6%	NC	de Carvalho et al. [66]
Chile	146	0%		10.5%	SV	Fardella et al. [67]
China	460	0.2%	NC	0.2%	NC	Wang et al. [78]
Czech Republic	174	6.5%	SV, NC	5.1%	NC	Kotaska et al. [68]
Croatia	186	5.9%	SV, NC	0%		Dumic et al. [69]
Denmark	136	2.2%	NC	4.4%	NC	Ohlsson et al. [70]
France^a^	322	3.6%	NC	55.9%	NC	Bidet et al. [23]
Finland	156	0%		2.6%	NC	Jaaskelainen et al. [19]
Germany	310	2.6%	SV, NC	2.9%	NC	Krone et al. [37]
Greece	222	11.3%	SV, NC	41.1%	NC	Dracopoulou-Vabouli et al. [71]
Italy	146	2.7%	NC	11%	NC	Carrera et al. [18]
India^b^	124	46%	SV	ND		Marumudi et al. [72]
Japan^c,d^	30	1.5 %	SV, NC	ND		Kashimada et al. [73]
Macedonia	122	19.7%	SV, NC	2.1%	NC	Anastasovska et al. [63]
Mexico	94	8.5%	SV, NC	8.5%	NC	Ordonez Sanchez et al. [74]
Netherlands	396	0.3%	NC	2.2%	NC	Stikkelbroeck et al. [14]
Romania	66	19.7%	SV	0%		Grigorescu-Sido et al. [75]
Serbia	122	13%	SV, NC	4.6%	NC, SV	Milacic et al. [62]
Spain	58	2.6%	NC	15.8%	NC	Ezquieta et al. [76]
Sweden	400	1.6%	NC	5.7%	NC	Wedell et al. [77]
USA	3005	2.6 %	SV, NC	23.9%	NC	New et al. [45]

*ND* not done

^a^Cohort consist of women with NC only

^b^Only investigating classic CAH

^c^Patients detected on neonatal screening

^d^In NC patients clitoromegaly was noted

## Clinical presentation and genotype/phenotype correlation

Although a good genotype–phenotype correlation has been established in up to 95% of patients with CAH [31, 45, 52], many outliers have been described, of which a significant number is due to the P30L mutation.

The enzyme activity is quite high in individuals with P30L mutations and mild form of CAH would be expected in all patients carrying it independently of the mutation on the other allele. Mild form is indeed the most frequent or the only presentation of P30L mutation in many; however, more severe forms are also present in a significant number of patients in other populations (Table 1) [33, 39, 45].

The NC form of CAH was initially called late-onset as clinical presentation was observed in adolescents and adults [38, 81, 82]. Presentation of NC CAH is subtle and diagnosis requires different diagnostic tests to exclude other metabolic problems. Moreover, clinical expression of NC CAH is variable in patients carrying the same mutation [1, 20, 45, 65]. In patients with NC CAH predominant signs are those of mild androgen excess. Therefore, in females the diagnosis is made mostly in late childhood, adolescence, or young adulthood due to symptoms as premature pubarche, acne, hirsutism, male-pattern alopecia, polycystic ovary syndrome (PCOS), and subfertility [11, 65, 79]. However, there are females with minimal or no symptoms, even after a long life [83]. Despite of similar 17OHP levels in patients with other mild mutations, NC patients with P30L mutation can show stronger virilization with clitoromegaly and advanced bone maturation [84]. Although I172N mutation is considered typically associated with SV, it seems that unlike other mild mutations, P30L mutation generates a continuum of phenotypes between NC and SV as well as a typical SV [33, 45, 85]. In a multinational study of 1507 families with CAH, SV form of CAH was found in 17/74 patients having P30L mutation (23%) [45]. Even 66% of patients with P30L mutation express unexpected virilization requiring extensive reconstructive surgery [86]. Moreover, in a study of a large cohort of patients with 21OHD of Greek origin, the phenotype of P30L mutation was equally distributed between SV and NC (19.1% vs 21.4%) [71]. The genotype/phenotype concordance in this study decreased as the severity of the disease diminished. Risk for short stature should be taken into consideration since NC and even SV sometimes are diagnosed late [87, 88], although in the majority final height was within the normal range [89–91].

In a Central European study the major genotype–phenotype discrepancies were detected for P30L and I172N mutation [80]. Similar findings have been confirmed in other studies [52, 69, 92]. In a study of 400 families in Argentina, P30L mutation, although rare (0.6%), the SV to NC phenotype ratio was 1:1 [16]. Moreover, in countries with high prevalence of the P30L mutation, in either a homozygous or compound heterozygote state, patients are prone to have increased virilization and fertility issues subsequently placing them in-between the NC and SV forms or even pure SV form [37, 40]. In the Republic of North Macedonia, where the prevalence of P30L mutation is among the highest in the world, the number of patients with SV form is very high [63]. Even when it is not a SV form, the clinical manifestation is with stronger signs of virilization, earlier adrenarche, clitoromegaly, and some patients require higher doses of glucocorticoids compared with other patients with NC form [55, 66, 93]. Moreover, genotypes P30L/I2 splice, P30L/Q318X, and P30L/8Δbp are especially associated with SV form of CAH (Table 2) [45]. Classical presentation of SV in two sisters with clitoromegaly, no breast development, and severely impaired fertility having P30L/I172N genotype has recently been described [40].

**Table 2:** Influence of different genotypes containing P30L mutation on the phenotype

Genotype	SV	NC	SW
P30L/del	7.2%	86.6%	7.2%
P30L/P30L	32.3%	66.7%	0%
P30L/I2 splice	65.2%	17.4%	17.4%
P30L/8Δbp	50%	50%	0%
P30L/I172N	22.2%	77.8%	0%
P30L/V281L	12.5%	87.5%	0%
P30L/Q318X	60%	40%	0%
P30L/R356W	33.3%	66.7%	0%

Data extracted from New et al. [45]

SV phenotype in girls is easier to recognize due to clitoromegaly, or severe atypical genital in newborn girls, but in boys the genital pigmentation might be missed, and early pubarche and advanced penile growth may be the first signs [41, 71, 86]. If not treated with glucocorticoids SV progresses steadily during childhood causing early puberty, short adult stature, and fertility issues in both genders including testicular adrenal rest tumors (TARTs) in men.

Thus, it might be advisable to be very cautious with the interpretation of the results of neonatal screening when P30L mutation is detected, since it might be the first sign of the SV phenotype which may manifest clinically later in childhood. Moreover, virilized girls without SW and fast growing boys with advanced bone age may not always have I172N but P30L mutation as shown in different studies [11, 24, 39, 73, 86].

## Diagnosis

The diagnosis of classical forms of 21OHD is based upon the clinical picture, blood electrolyte analysis, 17OHP levels, and androgens including testosterone, dehydroepiandrosterone sulfate (DHEAS), and androstenedione. Most patients have a basal morning 17OHP values above 30 nmol/L. However, some patients with suspected NC CAH may have lower basal 17OHP levels and a level between 6 and 30 nmol/L could warrant an ACTH-stimulation test [20, 41, 82, 94]. Levels above 30 nmol/L on ACTH-stimulation test, which is the golden standard for diagnosis of 21OHD, are considered diagnostic [22, 28, 41, 95]. Severe *CYP21A2* mutations have higher 17OHP levels both basal and post ACTH-stimulation [23]. Measurement of progesterone, 17-hydroxypregnenolone, 11-deoxycortisol, DHEAS, deoxycorticosterone, and androstenedione may be warranted in order to distinguish other forms of disturbed steroidogenesis. There are limited data on the biochemical parameters in patients with P30L mutation but these show similar 17OHP and testosterone levels as those with other mild mutations and cannot predict the severity of the clinical presentation [53, 96, 97]. Due to late diagnosis of NC and even SV forms, special attention should be given to children who grow faster than expected during early years of life, and follow them thoroughly for signs of early puberty. Bone maturation in these patients is of utmost importance for the diagnosis and follow-up [98]. Differences in the time of presentation and speed of progression in patients with P30L mutation remain to be elucidated. It should be noted that the different national neonatal screening programs are developed to diagnose classic CAH, and many NC newborns will not be detected if additional molecular testing is not applied as a second tier [28, 99]. It might be advisable to preform strict follow-up even in newborns with no symptoms where P30L mutation is detected.

## Therapy and follow-up

The goals of therapy in SV and NC forms, including patients with P30L mutation, are to substitute cortisol (especially in SV), reverse hyperandrogenism to ensure normal growth and timely puberty, preserve fertility as well as to avoid the long-term complications [100–102]. Hydrocortisone is the drug of choice in newborn and children with confirmed SV form [103]. However, appropriate glucocorticoid regimen (hydrocortisone, prednisolone, dexamethasone, or combinations) with or without mineralocorticoid therapy in adults is still uncertain [104–106]. Some SV patients may benefit from adding mineralocorticoids based on the studies showing higher plasma renin activity in SV patients including patients carrying P30L mutation. It is due to accumulation of some steroid precursors which can cause, especially in poorly controlled patients, aldosterone mediated transactivation of the human mineralocorticoid receptor. [107–109]. Adding mineralocorticoids in SV patients may provide decreased renin activity and may enable decrease of glucocorticoid dose. Mineralocorticoids have occasionally been used in NC CAH as well [110–113]. Therapy should be carefully tapered according to the growth pattern and hormonal results. There are trials to simplify frequent blood sampling with adrenal specific androgens measurements in saliva, hair, or urine samples [114–117]. Therapy changes with aging of the patients, fertility treatment is necessary in some females with CAH, both classic and nonclassic and men who develop TARTs, but also treatment of late complications of the disease and supraphysiological glucocorticoid supplementation [3, 81, 82, 111].

Therapy in patients with P30L mutations depends on the clinical picture, and consist mostly of improving symptoms, not biochemical findings. Achieving normal final height should be among the main goals. Transition from pediatric to adult care is of utmost importance since many patients could be lost from follow-up during this period [100, 118–120].

## Somatic outcomes

### Growth and puberty

Growth is affected in both females and males with CAH. Early prepubertal growth spurt due to early puberty is typical. Final height was affected (−2.5 SD) in earlier works, and significantly less (−1.0 SD for classic and −0.4 SD for NC form) in later works [91, 121–123], probably due to improved therapy and compliance. Hyperandrogenism can result in early closure of epiphyses. However, supraphysiological glucocorticoid supplementation may also lead to short adult height [122]. Close follow-up and fine tapering of therapy can improve the adult height, especially if the bone age advancement is detected before 8 years of age [3, 91, 124]. In SV form growth in early childhood in females is normal; however, in boys it is significantly faster and ends within 0.5 SD of target height [121]. The shorter stature in SV form is due to the early puberty, advanced bone age, and lack of important pubertal growth spurt [124, 125]. A high hydrocortisone dose during puberty may affect growth due to deterioration of the metabolic control [126–128]. Growth in NC form is often below the target height but within the normal range [96, 129, 130]. Careful tapering of therapy might provide height within the normal range and within the genetic potential.

There are no specific data on the influence of different mutations, yet the growth in patients with P30L mutation is expected to be affected mostly in males with delayed diagnosis of the SV form, and females with early puberty [131].

Puberty in patients with 21OHD occurs earlier compared with age-related peers [129, 132]. It occurs earlier in SW form (9.3 years on average in males and 9.2 years in females) compared with the age-related healthy population. In SV and NC form it occurred on average at ~8.5 years and at ~10 years in males and females, respectively [84, 128, 130]. Central precocious puberty in CAH is rare [41, 133]. There is no specific study but P30L should influence puberty onset causing earlier puberty and may induce secondary central precocious puberty in individuals with the SV form [84, 133]. In females, early puberty would be accompanied with clitoromegaly and impaired breast development [40, 134].

### Fertility

Fertility in females with all forms of CAH can be impaired, despite the advances in different therapeutic methods. The role of hyperandrogenism as a cause of impaired fertility has been extensively studied [135–137]. In females with SW form spontaneous fertility in older studies has been reported in only 2.5% and in SV 38% [138]. Additional 2% conceive by assisted reproductive technology. Other studies show higher but still low-fertility rate since it has been established that elevated circulating adrenal androgens and elevated serum progesterone concentrations may hinder ovulation and embrio implantation [139–141]. However, all agree that some additional factors contribute to the low reproductive rate such as the decreased sexual activity, higher sexual distress, higher prevalence of homosexuality or bisexuality, and unwillingness to pursue motherhood [136, 142–145]. Due to individualized fertility therapy, newer studies show much higher pregnancy rates in women with CAH approaching the fertility rate in the general population [81, 140]. Women with SV usually seek motherhood six times more frequently compared with those with SW [146]. Women with NC CAH conceive spontaneously in 57.2% [147]. Pregnancy in these women is normal with an outcome similar to the general population [136, 148]. There are no detailed data on fertility issues in patients with P30L mutation except the report of two female patients with P30L mutation and SV phenotype requiring genital surgery and several artificial assisted reproduction cycles [40]. In populations with a high prevalence of NC CAH, many females are diagnosed as PCOS [149–151]. NC CAH needs to be excluded before diagnosing PCOS [22, 82, 152, 153]. In one study of hirsute women 10% had NC CAH, some of them with P30L mutation [151]. Whether infertility issues are more frequent, or if they are more difficult to treat in the P30L mutation group remains to be elucidated in future studies.

Fertility issues in males with CAH are mostly due to TARTs. TARTs are common and appear in 40–94% in males, most commonly in severe forms of CAH [154–157]. They appear earlier and more frequently in patients with a poor metabolic control. TARTs have been described in patients with P30L even in childhood [158, 159]. Spermatogenesis in males with CAH is impaired and deteriorates with the age; however, associated obesity, common in older patients with CAH, might contribute as well [154, 156]. Still fertility was not decreased in 17 males with NC phenotype (none with P30L) or in 12 males with P30L compared with 1700 and 1200 matched controls, respectively [160]. Interestingly, in 221 males with 21OHD studied, only those born before the introduction of neonatal screening had impaired fertility [160]. Thus, early diagnosis may improve fertility in males with 21OHD [3, 160, 161].

Prenatal diagnosis in women with CAH is compromised by the possible genotype/phenotype discordance, especially when mutations causing adverse phenotypes such as P30L are detected [150]. Carriers of mild mutations might end up with unexpectedly high incidence of offspring with SW or SV form of the disease [162].

### Metabolic and cardiovascular outcomes

Therapy with glucocorticoids and androgen control influence metabolic status and outcome in all patients with CAH [163–166]. Long-term glucocorticoid replacement may cause abdominal obesity and hypertension with an onset even in youngsters [131, 167–169]. Obesity is frequently associated with high CRP levels, hypercholesterolemia, hyperlipidemia, insulin resistance, diabetes, high leptin, and low adiponectin levels causing a common metabolic syndrome in ~20% of patients with a cardiovascular risk independent of mutations [79, 123, 167, 170]. Very few studies analyzed cardiovascular outcomes according to the genotype [111, 123]. Interestingly, in the population study by Falhammar et al. different mutations had different risk of cardiovascular events [164]. Males with P30L mutation had one of the highest risk for any cardiometabolic condition and obesity as well as a tendency to increased risk for obstructive sleep apnea [164]. However, females with the P30L mutation had no cardiometabolic risk in this study but it should be noted that the number of studied individuals with this mutation was relatively low (*n* = 24). The metabolic issues were still present in those patients with CAH who were born after the introduction of neonatal screening [164]. Thus, cardiometabolic risk should be carefully monitored in patients with CAH. In fact, mortality has been shown to be increased in CAH (2.3 higher in males and 3.5 times in females compared with matched controls), of which a significant part was cardiovascular mortality [171].

### Bone health

Decreased bone mineral density and more fractures have been shown in some studies of CAH, but was absent in others [172–178]. Bone mineral density and fractures in patients with P30L mutations have not been studied in detail.

### Autoimmune diseases

Autoimmune diseases have recently been found to be more frequent in patients with CAH, and their prevalence increases with age. Compared with controls (*n* = 2900), those with P30L mutations (*n* = 29) had a tendency to develop more autoimmune disorders in general and especially rheumatoid arthritis [179]. Whether the onset of glucocortiocoid treatment and the duration of therapy have an immunomodulating effect remains to be elucidated [180].

## Mental outcomes

Engberg et al. analyzed psychiatric diagnoses in 335 women with CAH compared with 33,500 matched controls [181]. They found that psychiatric diagnoses in general and substance abuse were more common in women with CAH. Interestingly, patients with P30L mutation had much more psychotic disorders and personality disorders in the age group >18 years compared with the carriers of other mutations [181]. Psychotic disorders were increased in both female and male patients with P30L mutation, especially in those born before the neonatal screening [182], with personality disorders being more frequent in women with the P30L mutation [181]. In both genders with 21OHD, alcohol misuse was increased and so were also suicidal attempts in males. However, none in the P30L group has been diagnosed with alcohol misuse or attempted suicide [181, 182]. Similar findings were found in 226 individuals with CAH (almost all females) were psychiatric disorders and suicide attempts were more common than in the general population [166]. Although the genotypes were not described in the latter study it can be suspected that very few had P30L mutations. Whether late diagnosis, glucocorticoid therapy, and/or the androgen exposure contributes to the increased prevalence of psychiatric disorders in CAH, especially in women, remains to be elucidated [182, 183]. Since the level of hyperandrogenism has generally been associated with the alcohol and other substances abuse [184], and patients with P30L are more hyperandrogenic compared with the carriers of other mild mutations, it would be useful with larger studies to investigate addictions and psychiatric issues in this group [183].

### Quality of life

Having in mind the complexity of CAH and its complications, it has to be expected that patients with CAH will have affected quality of life (QoL) [11, 185]. Life-long therapy, frequent controls, additional issues as the patient grows, necessary interventions as well as under- or overtreatment and poor metabolic control leading to frequent sick-leave in CAH may result in lower social integration, education, self-confidence, employment, and lower QoL [186, 187]. Late diagnosis is associated with depression and decreased self-control. Many women with CAH are not satisfied with their sexual life and have later sexual debut or complete lack of sexual activity [141, 145]. Males with CAH had impaired sexual well-being in one study [188], but not in another study [186]. The overall psychosexual aspects of life were affected in these patients with later sexual debut, fewer pregnancies and children, and an increased incidence of homosexuality in women [189, 190]. In a QoL study from Norway including 72 adult participants with CAH impaired general health perception, vitality and working ability were found [191]. QoL was correlated to the severity of the mutations [5]. A recent systematic review reported increased psychological and psychiatric issues, impaired QoL, together with reduced satisfaction with reproductive health and sexual function in male with CAH [192]. QoL in patients with SV and NC forms can be similar to the controls as shown in patients diagnosed after the introduction of neonatal screening, probably due to the timely and more sophisticated treatment [154, 186].

## Conclusion

Patients with P30L mutations have not been studied extensively. The ethnic variability is wide and P30L mutation affects mostly people from Central Europe, Balkan countries, and Mexico. P30L confers 20–60% 21-hydroxylase activity. However, P30L mutation induces a more severe clinical virilization than the typical NC CAH and clinical presentation is a continuum between NC and SV phenotype. Studies of the structure of the mutated enzyme do not completely explain the discrepancy between the preserved enzyme activity and the phenotype. Therefore, the reclassification of this mutation as mild should be reconsidered. Long-term outcome data in patients with P30L are limited, but some issues such as psychiatric disorders may be more frequent in this group compared with the other mild mutations. Further studies of the genotype/phenotype variations in P30L careers, long-term outcomes, and treatment options are warranted.
